# The Role of Acculturative Stress, Emotion Dysregulation, and Escape Motivation in Problem Gambling Among Immigrants in Australia

**DOI:** 10.1007/s10899-026-10503-5

**Published:** 2026-04-22

**Authors:** Carla Virdis, Srivaasavi Sivasankar, Mal Flack, Kim M Caudwell

**Affiliations:** Discipline of Psychology, Faculty of Health, Charles Darwin University, Casuarina, Australia

## Abstract

Acculturative stress is a known risk factor for problem gambling among immigrant populations, but the psychological mechanisms underlying this relationship are not fully understood. This study tested a conceptual model in which the association between acculturative stress and problem gambling severity is explained through the sequential roles of emotion dysregulation and escape motivation. A sample of 70 first- and second-generation Australian immigrants completed an online survey measuring acculturative stress, emotion dysregulation, escape motivation, and problem gambling severity. Correlation analyses revealed significant positive intercorrelations among all variables. Hierarchical regression analysis showed that while acculturative stress was a significant initial predictor of problem gambling, its effect was fully attenuated when emotion dysregulation was added to the model. The addition of the escape motivation led to a further significant increase in explained variance, emerging as the strongest unique predictor in the final model. The findings support a pathway where acculturative stress contributes to emotion dysregulation, which in turn fosters gambling to escape negative affect, ultimately leading to greater gambling severity. Clinical implications suggest that interventions for immigrant gamblers should address acculturative stress, emotion regulation skills and escape motives.

## Introduction

A robust body of research establishes a direct link between stress exposure and the aetiology and maintenance of problem gambling (Elman et al., 2010; Hwang & Ting, 2008; Jacoby et al., 2013). Daily stressors can trigger gambling urges, precipitating and sustaining problem gambling (Elman et al., 2010). For immigrant communities, gambling behaviours and vulnerabilities are shaped by a confluence of factors, including cultural norms that may normalise gambling as a social activity, experiences of social isolation or discrimination post-migration, and the use of gambling as a coping mechanism for stressors related to acculturation and settlement (Luo & Ferguson, 2017). The intersection of these enabling and motivational factors creates a unique risk profile for problem gambling within immigrant populations (Luo & Ferguson, 2017). Further, problem gambling risk unfolds within an environment shaped by commercial, legislative, and socio-cultural factors, and effective gambling harm prevention requires the implementation of population-based policy action, particularly for populations facing unique social and cultural vulnerabilities (Wardle et al., 2019).

Problem gambling broadly describes a variety of gambling behaviours which can lead to harm to gamblers, their families and the community (Delfabbro, 2013). In 2022, in Australia, 46% of all gamblers were classified as being at some level of harm using the Problem Gambling Severity Index, a common measure used to assess gamblers’ risk of harm (Australian Gambling Research Centre, 2023). However, despite lower gambling participation overall, the risk of harm was higher among individuals with non-Australian backgrounds, who may experience additional vulnerabilities such as acculturative stress, social isolation, language barriers, and limited access to culturally appropriate gambling support services (Fogarty, 2017; Suomi et al., 2025). A public health perspective emphasises that the harms from gambling extend far beyond those with a clinical diagnosis of Gambling Disorder, affecting a much larger segment of the population and generating high social costs (Wardle et al., 2019). For immigrant populations, these harms may be compounded by barriers to accessing culturally appropriate information and support, as well as different cultural understandings of gambling and problem gambling that can limit help-seeking and early intervention (Fogarty, 2017).

Among immigrants, stress plays a critical role in the development of addictive behaviours, including Gambling Disorder, and its impact on psychological well-being appears to be a more potent determinant than cultural orientation itself (Hwang & Ting, 2008; Jacoby et al., 2013). As Hwang and Ting (2008) found, regardless of an individual’s level of ethnic identity or acculturation, it is the degree of stress experienced during the acculturative process that most strongly predicts adverse outcomes like psychological distress, depression, and problem gambling. Acculturative stress can arise from the psychological challenges of navigating and adapting to a different cultural context, including navigating conflicts, discrimination, and cultural loss and can be moderated by the availability of resources such as effective coping strategies, robust social support networks, and proficiency in the host culture’s language (Berry, 2005; Sam & Berry, 2010). Studies with immigrants in Germany and Sweden corroborated that acculturative stress is a significant predictor of both the severity of problem gambling symptoms, and cravings to gamble (Jacoby et al., 2013; Nilsson et al., 2024). This suggests that for immigrants, gambling may function as a maladaptive coping mechanism to escape or regulate the negative emotional states generated by the persistent and unique challenges of acculturation.

Several etiological models have attempted to explain problem gambling, integrating biological, psychological, and social factors (Blaszczynski & Nower, 2002; Jacobs, 1986; Sharpe, 2002). Early foundational work by Jacobs (1986) proposed a general theory of addictions, where a predisposing physiological state, characterised by abnormal levels of arousal and intense emotional reactions, combines with the psychological consequences of unresolved childhood adversity. According to this view, individuals are driven towards addictive behaviours such as gambling as a maladaptive form of self-regulation, seeking to modulate their distressing internal state (Jacobs, 1986). Building upon this foundation, Blaszczynski and Nower (2002) introduced a more nuanced pathways model, which identifies distinct subgroups of pathological gamblers. In the pathways model, the emotionally vulnerable gamblers comprise individuals who are characterised by a history of adverse early life experiences, pre-existing affective disorders (such as anxiety or depression), and deficient coping and problem-solving skills. Emotionally vulnerable gamblers gamble to modulate their negative emotional states and to escape, and the addictive behaviour serves as a maladaptive cognitive-behavioural strategy (Blaszczynski & Nower, 2002; Nower et al., 2022). This pattern of avoidance and emotional regulation significantly heightens their vulnerability to developing and maintaining addiction patterns (Blaszczynski & Nower, 2002).

Empirical research consistently demonstrates that individuals with problem gambling exhibit significantly greater difficulties across multiple domains of emotion regulation compared to healthy controls (Lee et al., 2024; Williams et al., 2012). Williams et al. (2012) found that people experiencing problem gambling display a reduced capacity for cognitive reappraisal, lower emotional clarity, heightened impulsivity, and less acceptance of their emotions, even when controlling for symptoms of anxiety, depression, and stress. These emotion regulation difficulties are understood as a transdiagnostic risk factor, and contribute to the aetiology and maintenance of diverse psychological disorders, including depression, anxiety, and problem gambling (Cludius et al., 2020; Williams et al., 2012). The relationship between stress and emotion regulation is particularly salient, as stressful situations can directly compromise the cognitive resources necessary for effective regulatory control. This is corroborated by laboratory studies which show that acute stress directly impairs the ability to implement emotion regulation strategies (Raio et al., 2013). Moreover, this dynamic may be especially relevant for populations facing chronic stressors, such as acculturative stress, which is itself associated with emotional instability and difficulties in managing affective responses (Tummala-Narra, 2021). Consequently, for an individual with pre-existing regulatory difficulties, the experience of significant stress may overwhelm their coping capacity, thereby increasing reliance on maladaptive behaviours like gambling as a primary means of escape (Williams et al., 2012).

Current evidence suggests that problem gambling behaviour is driven by emotion-focused motivations, including the pursuit of excitement, the enhancement of self-esteem (ego-boosting), and, most critically, the desire to escape from aversive psychological states (Flack & Morris, 2015). Within this framework, the escape motivation is theorised to hold a central role in the maintenance of problem gambling (Caudwell et al., 2024; King et al., 2024). Escape refers to the use of gambling as a maladaptive cognitive-behavioural strategy to dissociate from negative emotions or stressful life circumstances (Flack & Morris, 2016). Escape is the behavioural manifestation of avoidance, a maladaptive emotion-regulation strategy that involves suppressing psychological experiences, such as thoughts, emotions, sensations, memories, and urges, while maintaining and exacerbating psychopathology (Aldao et al., 2010). Therefore, the escape motivation for gambling can be viewed as the behavioural manifestation and proximal correlate of difficulties with emotion regulation (Caudwell et al., 2024; King et al., 2024). The escape outcome expectancy has been empirically found to explain and moderate the relationship between emotion dysregulation and problematic gambling behaviour, by allowing the avoidance of distressing emotions as they arise (Alaba-Ekpo et al., 2024; Lee et al., 2024; Richardson et al., 2024). Emerging research with immigrant populations supports the notion that acculturative stress and psychological distress provide contexts in which individuals may resort to gambling for emotional relief. For example, Keovisai and Kim (2019) found that cultural displacement and acculturative experiences shaped gambling motives among Chinese immigrants in the U.S., implicating stress and coping processes in gambling engagement. Similarly, a phenomenological study by Kang and Shin (2019) with immigrant workers in Korea with various backgrounds highlights how settlement stress and marginalisation relate to problem gambling, consistent with models of emotion dysregulation and escape expectancy.

In summary, the convergence of significant stress, difficulties in emotion regulation, and the subsequent reliance on gambling as a maladaptive avoidance strategy creates a synergistic effect, making these individuals significantly more vulnerable to developing and maintaining problem gambling and experiencing associated harm (Blaszczynski & Nower, 2002; Williams et al., 2012). However, whilst it is known that stress contributes to substance use disorders and behavioural addictions by impairing emotion regulation and promoting maladaptive coping strategies like gambling for relief, the specific role of acculturative stress remains unclear.

To address this gap, the present study examines the role of emotion regulation difficulties and escape in problem gambling (Lee et al., 2024), where acculturative stress serves as an upstream vulnerability factor that contributes to emotion dysregulation. This dysregulation, in turn, is hypothesised to increase motivation to gamble as a form of cognitive escape, ultimately leading to greater problem gambling severity. Under this conceptualisation, the maladaptive coping mechanism that involves gambling as a means to alleviate negative emotional states is akin to the need for escape, where gamblers are vulnerable by nature due to their background, and have limited coping skills (Caudwell et al., 2024; Nower et al., 2022). The present study aims to expand on previous literature by investigating the relationship between acculturative stress and problem gambling, by examining the role of emotion regulation difficulties (or emotion dysregulation) and escape outcome expectancy in accounting for this relationship. This study focuses on an understudied population, comprised of first- and second-generation immigrants in Australia who gamble. This population may face significant acculturative stress and emotion regulation difficulties and may be vulnerable to gambling-related harm.

Specifically, it is hypothesised that:

- *H*1. Acculturative emotion dysregulation, escape outcome expectancy (i.e., gambling to avoid aversive moods), and problem gambling severity will be positively correlated.
- *H*2. Acculturative stress will have a predictive effect on problem gambling severity, which will attenuate when emotion dysregulation is added to a hierarchical regression model.
- *H*3. Escape motives will emerge as a unique predictor of problem gambling severity, accounting for acculturative stress and emotion dysregulation.

## Method

### Participants and Procedure

This study employed a cross-sectional design and involved the use of an online self-report survey, assessing problem gambling, acculturative stress, emotion regulation, and escape motivation for gambling. Participants were recruited via social media platforms (e.g., Facebook). Inclusion criteria were as follows: (1) aged 18 years or older, (2) currently residing in Australia, (3) a first- or second-generation Australian immigrant, and (4) having gambled at least once in the past 12 months. A priori power analysis for hierarchical multiple regression indicated that a minimum of 55 participants would be needed to achieve 80% power, with an alpha level of 0.05 (Cohen, 2013; Soper, 2025). Based on literature findings, the power analysis was based on medium effect sizes among acculturative stress, emotion dysregulation, escape motivation for gambling, and problem gambling severity (Jacoby et al., 2013; Lee et al., 2024; Williams et al., 2012).

A total of 70 adults, either first- or second-generation Australian immigrants, completed the online survey. The sample had a mean age of 33.2 years (SD = 15.47), with ages ranging from 18 to 88. The gender distribution was 55.7% male (*n* = 39) and 44.3% female (*n* = 31). All participants were currently residing in Australia, with the highest representation from Victoria (28.6%, *n* = 20), New South Wales (21.4%, *n* = 15), and South Australia (24.3%, *n* = 17). Further, participants self-reported their cultural background, which was categorised according to the United Nations Statistics Division scheme. The regional distribution was as follows: Asia (42.9%, *n* = 30), Europe (12.9%, *n* = 9), Oceania (34.3%, *n* = 24), Africa (1.4%, *n* = 1), USA and Canada (7.1%, *n* = 5) and Latin America and the Caribbean (1.4%, *n* = 1). The majority of the participants identified as first-generation Australian immigrants (64.3%, *n* = 45) and 35.7% (*n* = 25) as second-generation Australian immigrants.

Based on the percentage of participants who engaged in each activity at least once a year, playing Bingo or buying raffle tickets (50.0%) and playing cards for money (50.0%) were the most common forms of gambling. This was followed by betting on casino Table (48.6%), sports betting (42.9%), playing pokies at pubs/clubs (38.6%) or at the casino (38.6%) and betting on horse/dog races (31.4%).

Participants completed an online Qualtrics survey assessing acculturative stress, emotion dysregulation, escape motivation for gambling, and problem gambling (see Measures). All responses were anonymous and securely stored on Qualtrics servers. The survey required approximately 20 min to complete, after which participants could enter a draw to win one of four AU$25 eGift cards, via a separate survey to maintain anonymity. This study was classified as low-risk and received ethical approval from the Researchers’ University Human Research Ethics Committee (HREC: H23041). To address the potentially sensitive nature of the topics, participants were provided with mental health support resources.

## Measures

### Problem Gambling

The Problem Gambling Severity Index (PGSI) is a 9-item instrument designed to assess problem gambling behaviour over the past 12 months (Ferris & Wynne, 2001). Participants respond to items such as “Have you bet more than you could really afford to lose?” using a 4-point Likert scale ranging from 0 (never) to 3 (almost always). The majority of participants (37.1%) were classified as ‘non-problem’ or 15.7% ‘low risk’ (15.7%), whereas were 24.3% ‘moderate risk’, and 22.9% ‘high risk’. The total PGSI score was used in the analyses.

## Emotional Dysregulation

The Difficulties in Emotion Regulation Scale - short form (DERS-SF) is a transdiagnostic measure, evaluating emotion regulation deficits through self-reported perceptions, avoiding the reference to specific emotion regulation strategies (Gratz & Roemer, 2004). Participants rate items like “When I’m upset, I feel out of control” on a 5-point Likert scale ranging from 0 (almost never) to 5 (almost always). Total scores, calculated by summing all 24 items, reflect overall emotion dysregulation severity, with higher scores indicating greater difficulties. The DERS has shown strong reliability in prior research (Burton et al., 2022; Marchica et al., 2019).

## Escape Gambling Outcome Expectancy

The Gambling Outcome Expectancies Scale (GOES) is an 18-item multidimensional measure assessing motivations for gambling (Flack & Morris, 2015). The current study utilises the escape subscale (4 items), as it focuses on the anticipated relief from negative cognitive and emotional states through gambling. In this study, this subscale serves as an operational measure of the desire to use gambling as a maladaptive coping mechanism. Participants respond to items using a 6-point Likert scale ranging from 1 (strongly disagree) to 6 (strongly agree). The subscale scores are calculated by averaging item responses, with higher scores indicating greater endorsement of the escape motivation (Flack & Morris, 2015). The GOES has demonstrated sound factor structure (Flack & Stevens, 2019) and good internal consistency (Vaughan & Flack, 2022).

### Acculturative Stress

Social, Attitudinal, Familial, and Environmental Acculturative Stress Scale – short form (SAFE-13) is a 13-item self-report instrument designed to measure acculturative stress in multicultural populations (Mena et al., 1987). It assesses stress arising from social, attitudinal, familial, and environmental challenges related to cultural adaptation. Items include statements such as “I feel uncomfortable when others make jokes about or put down people of my ethnic background.” Participants rate each item on a 5-point Likert scale ranging from 1 (not stressful) to 5 (extremely stressful). Total scores are computed by summing all items, with higher scores indicating greater levels of acculturative stress. The SAFE-13 has demonstrated good internal consistency and construct validity across diverse ethnic groups (Suh et al., 2016).

## Results

### Data Preparation and Analytical Plan

Descriptive and inferential statistics were performed using SPSS, version 30.0. Variables of interest (SAFE, PGSI, DERS, and GOES-escape) were screened for outliers, skewness, and kurtosis. Based on Field (2017), all predictor variables met the criteria for acceptable skewness and kurtosis values ( < ± 2). An examination of Cook’s distance revealed no concerning influential multivariate outliers. The distribution of the residuals was examined visually using quantile-quantile (Q-Q) plots, and no substantial deviations from normality were observed (Field, 2017). A visual inspection of predicted versus residual values confirmed that the assumptions of linearity and homoscedasticity were met (Field, 2017).

The analyses first examined the bivariate relationships among all variables. To test the hypothesis that acculturative stress is a significant positive predictor of problem gambling severity, a hierarchical linear regression was conducted. Immigrant category (i.e., first- or second-generation immigrant) was entered as a control variable in the first step of the regression model. In the second step, acculturative stress was entered as a predictor. In the third step, emotion dysregulation was added to the model to determine if it attenuated the relationship between acculturative stress and problem gambling severity. Lastly, escape motivation for gambling was added to the model to evaluate its predictive power for problem gambling severity.

Prior to the main analysis, preliminary analyses were conducted to assess the reliability of the measures and their intercorrelations (Table 1). Internal consistency was excellent for the PGSI, SAFE, and GOES-Escape scales, and good for the DERS. Descriptive statistics are presented in Table 1.

**Table 1:** Descriptive Statistics and Correlations Between Variables of Interest

Variable	1	2	3	4	M	SD
1. SAFE	α = 0.93				49.74	16.58
2. DERS	0.34**	α = 0.88			43.23	11.30
3. Escape	0.37**	0.44**	α = 0.92		2.30	1.33
4. PGSI	0.33**	0.43**	0.60**	α = 0.91	4.43	5.92
5. Immigrant category	− 0.16	0.12	0.06	0.07	-	-

**p < .01, *p <. 05; Immigrant category code 0 = first generation, 1 = second generation. Cronbach’s α is presented along the principal diagonal

#### Hypothesis 1

Positive Intercorrelations Between Acculturative Stress, Problem Gambling, Emotion Dysregulation, and Escape Motivation for Gambling

The first hypothesis, concerning the relationships between acculturative stress, emotion regulation difficulties, escape motivation, and problem gambling, was examined using correlation analysis (see Table 1). The results supported H1, revealing significant positive intercorrelations among all variables. Problem gambling severity (PGSI) was significantly correlated with emotion regulation difficulties (DERS-SF), escape motives for gambling and acculturative stress (SAFE). Acculturative stress (SAFE) was significantly correlated with emotion dysregulation (DERS-SF) and escape motives. The strongest correlation was observed between escape motives and problem gambling (PGSI). All correlations were positive and of small to moderate magnitude, indicating that while related, each construct maintains discriminant validity.

#### **Hypotheses 2 and 3**

Hierarchical Regression Model Testing the Attenuation of Acculturative Stress and the Role of Emotion Dysregulation and Escape Motivation for Gambling in Predicting Problem Gambling.

To test the unique and sequential contributions of the predictor variables, a four-step hierarchical regression was conducted. This method is statistically robust for the sample size and allows for a clear test of the hypothesis that the relationship between acculturative stress and problem gambling would attenuate upon the introduction of the proposed mediators (emotion dysregulation and escape motivation). Problem gambling severity (PGSI) was entered as the dependent variable, and, following the inclusion of immigration category in the first block, the following steps were taken in sequence: acculturative stress (SAFE), emotion dysregulation (DERS-SF), and gambling motivation to escape (GOES-Escape). The first step of the regression analysis (immigration category) was non-significant, *F*(1, 68) = 0.31, *p* = .580, *R*² = 0.01. The second step showed that acculturative stress (SAFE) was a significant predictor of problem gambling, *F*(1, 67) = 8.00, *p* = .004, ∆*R*² = 0.12. At the third step, which introduced emotion dysregulation (DERS-SF) as a predictor, the model change was significant, *F*(1, 66) = 8.63, *p* = .005, ∆*R*² = 0.10. At the final step, where gambling motivation to escape (GOES-Escape) was added as a predictor, the model was significant, *F*(4,65) = 10.89, *p* < .001, *R*² = 0.40). In this step, the escape motivation (GOES-Escape) was a significant unique predictor of problem gambling; *F*(1, 65) = 19.26, *p* < .001, ∆*R*² = 0.17, while both acculturative stress and emotion dysregulation (DERS-SF) were not. Overall, the final model accounted for 40.1% of the variance in problem gambling (Table 2).

**Table 2:** Hierarchical Regression Analysis

Independent Variables	*R* ^2^	B	β	t	*p*
MODEL 1	0.01				
1. Immigration category		0.87	0.07	0.56	0.58
MODEL 2	0.12**				
1. Immigration category		1.53	0.12	1.07	0.29
2. Acculturative stress		0.12	0.35	3.00	0.004
MODEL 3	0.22**				
1. Immigration category		0.76	0.06	0.55	0.583
2. Acculturative stress		0.08	0.22	1.85	0.069
2. Emotion dysregulation		0.18	0.35	2.94	0.005
MODEL 4	0.40**				
1. Immigration category		0.38	0.03	0.31	0.756
2. Acculturative stress		0.03	0.09	0.86	0.400
3. Emotion dysregulation		0.09	0.18	1.60	0.114
4. Escape		2.17	0.49	4.39	< 0.001

**p < .01, *p <. 05

## Discussion

The present study examined the interrelationships between acculturative stress, emotion regulation, escape motivation, and problem gambling severity among first- and second-generation immigrants in Australia. The findings largely supported the hypotheses, revealing associations among all variables, and suggesting that acculturative stress significantly predicts problem gambling, but its influence is decreased when emotion regulation difficulties and escape motivation are accounted for.

The correlational analyses provided support for Hypothesis 1, confirming that acculturative stress, emotion dysregulation, escape motivation for gambling, and problem gambling severity are all positively interrelated. The pattern of small to moderate correlations indicates that these constructs are related yet distinct, suggesting good discriminant validity. Acculturative stress was significantly correlated with emotion dysregulation, escape motivation, and gambling severity, suggesting that stressful cultural adaptation experiences are linked with both emotional vulnerabilities and maladaptive coping behaviours. These findings align with prior evidence that chronic stress undermines emotion regulation capacity and fosters maladaptive behavioural regulation strategies such as gambling (Raio et al., 2013; Tummala-Narra, 2021). Consistent with expectations, emotion dysregulation showed significant, moderate positive associations with both problem gambling severity and escape motivation, replicating previous findings that deficits in emotion regulation are a key vulnerability for gambling to escape negative affect (Lee et al., 2024; Marchica et al., 2019). The strongest correlation was observed between escape motivation and problem gambling severity (PGSI), supporting the conceptualisation of escape as a behavioural expression of emotion regulation difficulties (Lee et al., 2024; Marchica et al., 2019). The associations are also aligned with the emotionally vulnerable pathway proposed by Blaszczynski and Nower (2002), where individuals high in both acculturative stress and dysregulation may be particularly prone to using gambling as a maladaptive coping strategy to escape aversive internal states. The present findings, therefore, situate acculturative stress within this framework as a contextual stressor that exacerbates emotion regulation difficulties, which in turn intensify the likelihood of gambling to cope.

### Hypothesis 2

posited that the association between acculturative stress and problem gambling severity would attenuate when emotion regulation was added to the model. As noted, acculturative stress was associated with problem gambling, which is consistent with previous research where acculturative stress has been identified as a significant contributing factor to gambling harm among immigrant groups (Hwang & Ting, 2008; Jacoby et al., 2013). However, when emotion regulation was introduced into the model, the relationship between acculturative stress and problem gambling attenuated to non-significance. This attenuation indicates that the influence of acculturative stress on gambling severity may not be direct but rather operates primarily through its impact on emotion regulation capacity. Therefore, this finding suggests emotion regulation may play an important role in the development and maintenance of problem gambling within the multicultural context. This notion also aligns with a robust literature identifying emotion dysregulation as a transdiagnostic factor of addictive behaviours (Buen & Flack, 2022; Cludius et al., 2020; Velotti et al., 2021). The present findings extend this literature by positioning emotion regulation as a significant psychological mechanism that may translate acculturative stress into maladaptive behaviours such as problem gambling.

The final hypothesis examined whether escape motivation would emerge as the dominant predictor of problem gambling. The findings revealed that the escape motivation was the unique predictor of problem gambling, accounting for the influence of acculturative stress and emotion regulation. This result suggests that escape motivation carries its own predictive power for problem gambling severity. This finding is consistent with, and extends, the recent work of Lee et al. (2024), who found that escape outcome expectancies partially mediated the relationship between emotion regulation and problem gambling. The present pattern of findings indicates that the anticipation that gambling will provide relief from undesirable emotional states remains a powerful maintaining factor in problem gambling. It is also plausible that the salience of role of gambling to escape may not only reflect the influence of acculturative stress and emotion regulation difficulties, but gambling to escape may also be an outcome of other stressors, such as exposure to trauma (Moore & Grubbs, 2021), homelessness (Vandenberg et al., 2022), and the negative consequences from problem gambling itself (Tseng et al., 2023).

### Practical and Clinical Implications: The Utility of the Escape Motive

The findings carry important practical implications, particularly concerning the applied use of the escape motivation as a measure for problem gambling risk in immigrant communities. Emotion dysregulation can be a complex psychological construct to assess in non-clinical settings and may not be directly targeted in brief interventions. In contrast, the escape motivation for gambling, as measured by the GOES-Escape subscale, is a more easily measurable construct. It captures a gambler’s explicit reason for engaging in the gambling behaviour. The fact that escape motivation emerged as a unique predictor suggests that screening for this specific motive could be a highly efficient way to identify individuals at heightened risk for problem gambling. In primary care, short screenings that include questions about gambling to escape or forget problems could help flag individuals in need of early intervention. Within migrant support services, incorporating culturally sensitive screening questions that assess gambling as a means of emotional escape may be particularly valuable, as these services are often the first point of contact for individuals experiencing acculturative stress, social isolation, and settlement-related distress (Hynie, 2018). Embedding such screening and targeted psychoeducation within settlement and community services aligns with calls to integrate mental health and coping-focused interventions into migrant care (Tummala-Narra, 2021).

Beyond screening, migrant support services are well-positioned to offer preventative and early-intervention supports that directly target stress and coping before gambling behaviour escalates. Evidence suggests that community-based and culturally responsive interventions, such as programs that provide social connection, language support, employment assistance, and psychoeducation about stress and coping, can significantly reduce psychological distress among immigrants (Hynie, 2018; Silove et al., 2017). Interventions that strengthen social support and promote adaptive coping have been shown to lessen the impact of acculturative stress and reduce reliance on avoidance-based strategies, particularly when delivered through trusted community organisations rather than specialist mental health settings (Schweitzer et al., 2006). Integrating stress-management skills, emotional literacy, and normalisation of acculturative challenges within migrant services may therefore reduce the need to seek emotional relief through maladaptive behaviours such as gambling, while also overcoming barriers related to stigma and limited access to formal mental health care (Hynie, 2018; Tummala-Narra, 2021).

### Importance, Limitations, and Future Directions

A significant aspect of this study is its focus on first- and second-generation immigrants, a vulnerable population that is both hard to reach and underrepresented in gambling research. Despite a nationwide recruitment campaign, the final sample size was modest, reflecting the considerable barriers to engaging culturally and linguistically diverse communities in research. These barriers can include stigma surrounding mental health and gambling, mistrust of academic institutions, language differences, cultural norms that discourage disclosing personal problems, and a simple lack of awareness of or access to research opportunities (Sam & Berry, 2010). The limited inclusion of these groups in research perpetuates health disparities and results in intervention strategies that are not culturally attuned or effective for all segments of the population (Pardhan et al., 2025). By specifically investigating the role of acculturative stress, this study moves beyond a one size fits all model of gambling aetiology and acknowledges the unique socio-cultural experiences that can shape gambling behaviour (Australian Gambling Research Centre, 2016). The findings provide a foundational knowledge base that can inform the development of targeted public health messages and culturally sensitive clinical guidelines for a vulnerable population that might otherwise be overlooked. Understanding that gambling in this context may be deeply intertwined with the stresses of migration and emotional coping can help clinicians, community workers, and policy makers approach prevention and treatment with greater empathy, cultural competence, and effectiveness (Australian Gambling Research Centre, 2016). This work is a necessary step towards reducing the gambling-related health inequity that disproportionately affects immigrant communities.

Several limitations of the present study must be acknowledged. First, the cross-sectional design precludes definitive causal inferences. While the data are consistent with a model where acculturative stress leads to dysregulation, which leads to gambling to escape, the reverse or bidirectional relationships are also plausible. For example, problem gambling can itself generate stress (financial, legal, relational) and impair emotion regulation. Longitudinal studies that track these variables over time, especially during the acculturation process, are essential to establish temporal precedence and confirm the proposed causal pathway. Second, the sample size, though adequate for the analyses conducted, was modest and may limit the generalisability of the findings. The sample was also self-selected, which may introduce bias. Future research should seek to replicate these findings with larger, more representative samples of immigrant gamblers from diverse cultural backgrounds. Third, the analytical approach, while appropriate for the sample size and data characteristics, provides evidence consistent with mediation but does not constitute a formal test of the indirect effects. A more powerful study with a larger sample would be required to employ mediation analysis techniques, which would allow for a direct statistical test of the proposed sequential pathway from acculturative stress to problem gambling via emotion dysregulation and escape motivation (Hayes, 2017). Fourth, the study relied solely on self-report measures, which are susceptible to social desirability bias. This is a particular concern in culturally diverse samples, where stigma surrounding gambling and mental health may influence the accuracy of self-disclosure (Australian Gambling Research Centre, 2016). Future studies could benefit from incorporating multi-method assessments, including behavioural tasks of emotion regulation or interviews with collateral informants.

Future research should also investigate whether escape motives operate differently across cultures and gambling modalities (e.g., online vs. land-based gambling), given evidence that motives vary across different cultures (Oei & Raylu, 2010) and activity types (Richardson et al., 2024). Moreover, the relatively high reported engagement in activities like playing bingo and poker machines, often perceived as low-stakes, passive, and repetitive, could be particularly conducive to a dissociative or escapist experience (Flack & Stevens, 2019). Future research should explore whether these activities are disproportionately used for emotional escape compared to other forms of gambling, which could allow for more precise public health messaging within immigrant communities about the risks of using any gambling form for mood regulation.

Finally, future research should explore potential protective factors that might buffer against this emotion dysregulation-led pathway. Factors such as strong ethnic identity, social support from both co-ethnic and host communities, and engagement in traditional or alternative coping practices may increase resilience to gambling risk in multicultural populations (Sam & Berry, 2010; Yoon et al., 2013). Investigating these resilience factors could provide a more detailed understanding of immigrant populations and inform strength-based intervention strategies. Additionally, qualitative research could offer rich and nuanced insights into the lived experience of acculturative stress and the personal meanings attached to gambling within immigrant communities, further guiding culturally grounded interventions.

## Conclusion

This study demonstrated that the relationship between acculturative stress and problem gambling severity in a sample of Australian immigrants is not direct but likely mediated by emotion dysregulation and escape motivation. While acculturative stress represents a vulnerability factor in multicultural populations, it is the difficulties in managing emotional distress that emerge following acculturative stress, and the reliance on maladaptive escape motives that more directly maintain problem gambling. Despite the challenges in recruiting from the Australian immigrant population, such research is essential for developing a more inclusive and comprehensive understanding of problem gambling. This study attempts to contribute to this critical evidence base, which is needed to inform strategies to improve wellbeing in multicultural communities where gambling is prevalent or perceived as a way of managing stress related to the migrant experience.
